# Supplementary material for: Observation of nonlinear response and Onsager regression in a photon Bose-Einstein condensate
Source: Nat Commun. 2024 Jun 3;15:4730. doi: 10.1038/s41467-024-49064-9 (PMC11148057; doi:10.1038/s41467-024-49064-9)
Supplement: Supplementary file 1 — Supplementary Information [file 41467_2024_49064_MOESM1_ESM.pdf]

Supplementary Information for
"Observation of Nonlinear Response and Onsager Regression in a Photon Bose-Einstein Condensate"

Alexander Sazhin [1], Vladimir N. Gladilin [2], Andris Erglis [3], Göran Hellmann [1], Frank Vewinger [1], Martin Weitz [1], Michiel Wouters [2], and Julian Schmitt [1]

[1] *Institut für Angewandte Physik, Universität Bonn, Wegelerstr. 8, 53115 Bonn, Germany*

[2] *TQC, Universiteit Antwerpen, Universiteitsplein 1, B-2610 Antwerpen, Belgium*

[3] *Physikalisches Institut, Albert-Ludwigs-Universität Freiburg, Hermann-Herder-Straße 3, 79104, Freiburg, Germany*

The Supplementary Information contains additional figures discussed in the Methods section of the paper by Sazhin et al., "Observation of Nonlinear Response and Onsager Regression in a Photon Bose-Einstein Condensate". Supplementary Fig. 1 shows a second-order correlation calibration measurement, Supplementary Fig. 2 gives theory plots showing that the grand canonical particle number fluctuation dynamics of the photon Bose-Einstein condensate are expected to be characterised by linear equations of motion. Finally, Supplementary Fig. 3 shows experimental time traces of the nonlinear BEC response to strong perturbations of increasing strength and characterises the accuracy of the linear and nonlinear fit models based on numerical data.

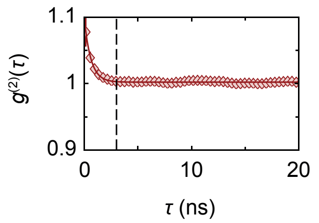

Supplementary Fig. 1. **Second-order correlations $g^{(2)}(\tau)$ measured for HeNe laser.** The measurement is used to benchmark the photomultiplier-based detection method of $g^{(2)}(\tau)$. The 'fake' bunching signal at $\tau < 3\,\mathrm{ns}$ (dashed line) is not caused by actual photon bunching; instead, it is attributed to electronic noise from the single-detector measurement system. The early-time data points are thus disregarded for the analysis of the photon dynamics. Solid line shows exponential fit with time constant of $0.6\,\mathrm{ns}$.

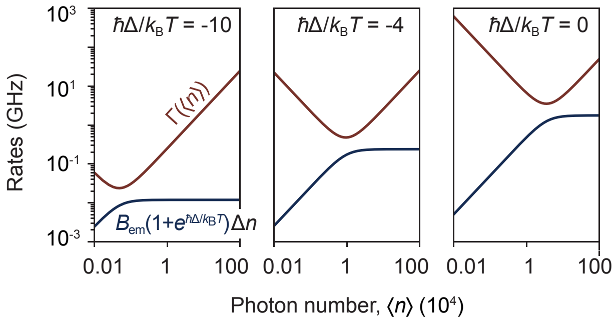

Supplementary Fig. 2. **Linearity of grand canonical number fluctuations.** Graphical comparison of rates from eq. (23) of the main text for a photon condensate subject to reservoir-induced fluctuations for dye-cavity detunings $\hbar\Delta/k_{\mathrm{B}}T = \{-10, -4, 0\}$. The linear fluctuation rate $\Gamma(\langle n\rangle)$ (red) exceeds the rate that would lead to nonlinear dynamics (blue) for all detunings and photon numbers. Correspondingly, the dynamics of reservoir-induced fluctuations are expected to be well described by linear equations, in agreement with the experiments.

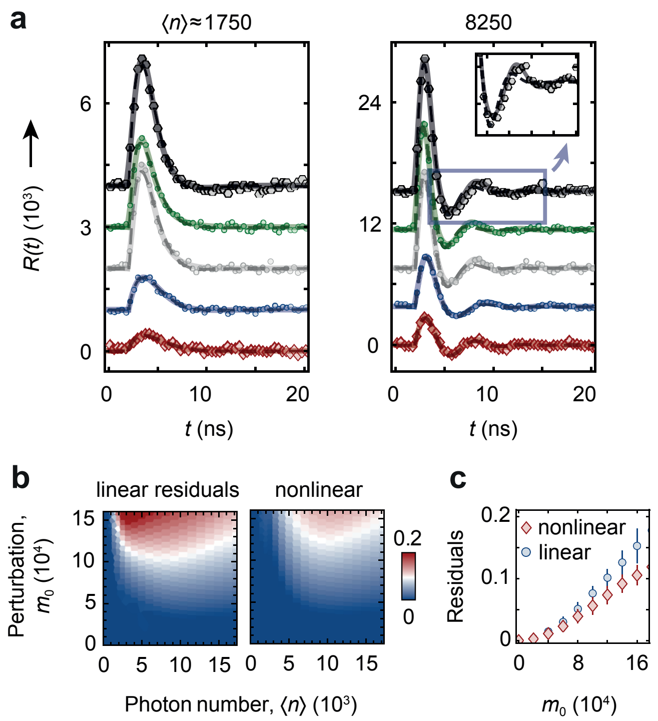

Supplementary Fig. 3. **Experimental time traces of nonlinear BEC response and accuracy of fit models. a**, Experimental time traces of condensate response for increasing perturbation strength (bottom to top; vertically shifted) for $\langle n \rangle \approx 1750$ (left panel, biexponential) and 8250 (right, oscillatory) along with fits. While at moderate perturbations the data is well described by both linear (dashed) and nonlinear (solid) fits, the nonlinear model matches the observed dynamics better for strong perturbations. **b**, Crossover from linear (blue) to nonlinear (red) photon dynamics versus initial perturbation strength m_0 and photon number $\langle n \rangle$ as obtained from numerical simulations; colours indicate fit residuals. The nonlinear model exhibits generally smaller residuals and therefore matches the numerical data better especially in the limit of large perturbations. **c**, Numerically calculated residuals versus m_0 averaged for measurements with $\langle n \rangle \geq 4000$, showing the qualitatively same splitting behaviour as seen experimentally, see Fig. 4c of the main text.
